# Dietary Quality Predicts Short-Term Change in Mobility Limitations in Older U.S. Adults

**DOI:** 10.1093/geroni/igaa057.773

**Published:** 2020-12-16

**Authors:** Nicholas Bishop, Sarah Ullevig, Krystle Zuniga, Kaipeng Wang, Julia Tucker

**Affiliations:** 1 Texas State University San Marcos, San Marcos, Texas, United States; 2 The University of Texas at San Antonio, San Antonio, Texas, United States; 3 The University of Texas at Austin, Dell Medical School, Austin, Texas, United States; 4 University of Denver, Denver, Colorado, United States; 5 Texas State University, New Braunfels, Texas, United States

## Abstract

Using a representative sample of older Americans, this project examines the association between dietary quality and short-term change in mobility limitations and whether this association differs for the food insecure or those receiving nutritional assistance. The sample was drawn from the 2012 Health and Retirement Study and 2013 Health Care and Nutrition Study and included 3,779 respondents representing a population of 37,217,566 adults aged 65 and older. Mobility limitations were operationalized as a log-transformed count of 11 indicators of limitation in physical mobility. Dietary quality was measured using the Alternative Healthy Eating Index-2010 (AHEI-2010) based on responses to a food frequency questionnaire. Food insecurity was a binary measure based on the USDA six-item short form. Nutritional assistance included receipt of supplemental food from sources such as food banks and/or reporting receipt of SNAP benefits (1=yes, 0=no). Autoregressive multiple regression was used to test whether AHEI-2010 predicted change in mobility limitations from 2012-2014 and whether food insecurity or receipt of supplemental food moderated this relationship. Around 10.7% of older adults were food insecure, and around 17.5% reported receipt of nutritional assistance. AHEI-2010 was associated with a slower decline in mobility limitations over the 2-year observational window (b=-0.014, SE=.003, p<.001), but food insecurity nor nutritional assistance were associated with changing mobility limitation, either directly or as moderators of the association between progressing limitations and AHEI-2010. These preliminary findings suggest dietary quality may be associated with disablement among older adults regardless of food environment.

